# Systematization of Nursing Care: how did the concept mature?

**DOI:** 10.1590/0034-7167-2022-0464

**Published:** 2023-08-07

**Authors:** Jackeline Felix de Souza, Kênia Rocha Leite Zaccaro, Ana Paula da Costa Lacerda Brandão, Cândida Caniçali Primo, Rosimere Ferreira Santana, Marcos Antônio Gomes Brandão

**Affiliations:** I Fundação Universidade Federal de Rondônia. Porto Velho, Rondônia, Brazil; II Universidade Federal do Rio de Janeiro. Rio de Janeiro, Rio de Janeiro, Brazil; III Universidade Federal do Espírito Santo. Vitória, Espírito Santo, Brazil; IV Universidade Federal Fluminense. Rio de Janeiro, Rio de Janeiro, Brazil

**Keywords:** Knowledge, Professional Practice, Nursing Process, Nursing, Concept Formation., Conocimiento, Práctica Professional, Proceso de Enfermería, Enfermería, Formación de Concepto., Conhecimento, Prática Profissional, Processo de Enfermagem, Enfermagem, Formação de Conceito

## Abstract

**Objectives:**

to analyze the Systematization of Nursing Care conceptual maturation from the perspective of pragmatic utility.

**Methods:**

a concept analysis study. The stages were: select the concept; elaborate analytical questions; comprehensively review the literature; and determine concept structural components. Sixty-one documents were analyzed after a search carried out until October 2019.

**Results:**

four temporal periods of contextual changes have occurred since the emergence of ideas of a systematization in the 1960s. This first lasted until 1990. It was followed by those from 1990 to 2002, from 2002 to 2009 and from 2009 onwards. Partial conceptual maturity was identified, operationalization over the years, based on multiple definitions, and, currently, a concept of Systematization of Nursing Care with managerial and organizational attributes.

**Conclusions:**

the concept of Systematization of Nursing Care is partially mature, presents multiple definitions, being operationalized in uncertain connections with other concepts.

## RESUMO

**Objetivos:**

analisar o amadurecimento conceitual de Sistematização da Assistência de Enfermagem na perspectiva da utilidade pragmática.

**Métodos:**

estudo de análise de conceito. As etapas foram: selecionar o conceito; elaborar questões analíticas; revisar a literatura de modo abrangente; e determinar componentes estruturais do conceito. Foram analisados 61 documentos após busca realizada até outubro de 2019.

**Resultados:**

quatro períodos temporais de mudanças contextuais ocorreram desde o surgimento das ideias de uma sistematização nos anos de 1960. Esse primeiro perdurou até 1990. Foi seguido pelos de 1990 a 2002, de 2002 a 2009 e a partir de 2009. Foi identificada parcial maturidade conceitual, operacionalização ao longo dos anos, com base em múltiplas definições, e, atualmente, um conceito de Sistematização da Assistência de Enfermagem com atributos gerenciais e organizativos.

**Conclusões:**

o conceito de Sistematização da Assistência de Enfermagem é parcialmente maduro, apresenta múltiplas definições, sendo operacionalizado em ligações incertas com outros conceitos.

## RESUMEN

**Objetivos:**

analizar la maduración conceptual de la Sistematización de los Cuidados de Enfermería en la perspectiva de la utilidad pragmática.

**Métodos:**

estudio de análisis de concepto. Los pasos fueron: seleccionar el concepto; elaborar preguntas analíticas; revisar exhaustivamente la literatura; y determinar componentes estructurales del concepto. Se analizaron 61 documentos luego de una búsqueda realizada hasta octubre de 2019.

**Resultados:**

ocurrieron cuatro períodos temporales de cambios contextuales desde el surgimiento de ideas de una sistematización en la década de 1960. Esta primera duró hasta 1990. Le siguieron las de 1990 a 2002, de 2002 a 2009 y de 2009 en adelante. Se identificó madurez conceptual parcial, operacionalización a lo largo de los años, a partir de múltiples definiciones, y, actualmente, un concepto de Sistematización de la Atención de Enfermería con atributos gerenciales y organizativo.

**Conclusiones:**

el concepto de Sistematización de la Atención de Enfermería está parcialmente maduro, tiene múltiples definiciones y se operativiza en conexiones inciertas con otros conceptos.

## INTRODUCTION

Inaccurate conceptions of the Systematization of Nursing Care (SNC) term can lead to mistaken or limited interpretations, influencing its conceptual application^(1)^. Such interpretations raise the growing interest of professionals and academics for the conceptual clarification of the term. However, debates and publications existing today tend not to precisely explain the SNC ontology or offer operational definitions^(2)^, except for some contributions already presented in manuscript in pre-print format.

In the contemporary professional context, nursing class entities and a network of researchers have used strategic actions to promote the SNC’s and the Nursing Process’ (NP) conceptual design. The Brazilian Association of Nursing (ABEn - *Associação Brasileira de Enfermagem*), a scientific body in the area, has for some years been including, in its documents, goals to encourage the debate on the SNC and the NP^(3)^. The Federal Council of Nursing (COFEN - *Conselho Federal de Enfermagem*), the body that regulates professional nursing practice in Brazil, established a working group to update the norm that regulates NP and SNC implementation in health units^(4)^.

In the academic context, for more than a decade, Brazilian researchers have shown the existence of different currents of thought for the mentioned terms, with the permanence of conceptual inaccuracies and terminological overlap in recent years^(5-10)^. Nursing Process Research Network (REPPE - *Rede de Pesquisa em Processo de Enfermagem*) researchers, when reflecting on the NP concept in Brazil, expressed the need to expand the SNC concept’s conceptual and operational definitions so that there is no overlap between its definition and that of other concepts^(11)^.

The issue that still represents a barrier to the advancement of knowledge on the subject refers to non-exploration of temporal origin and dynamics of overlapping SNC concept attributes, by most of available publications. Although there are already reflexive gains in the literature from the aggregation of evidence by reviewing the literature and qualitative research^(1-2,9-10,12)^, the temporal markers of conceptual evolution and SNC concept components still remain uncertain. Moreover, available research is insufficient to delineate or clarify the concepts and identify systematization meanings contained in original elements of nursing production, particularly between the 1970s and 1990s, a period prior to the first COFEN Resolution 272 of 2002 on the NCS^(13)^.

There is a tendency to recognize that the SNC is a Brazilian construct, which leads researchers to investigate the concept fundamentally in Brazilian literature. Despite this, there are potentially related constructs in other countries, such as the Professional Practice Model, which, to a certain extent, would be a possible model of professional practice with convergent attributes to that of the SNC. Thus, the challenge of advancing knowledge about the SNC construct and producing extrapolations and comparisons with other international concepts depends on concept analysis^(14)^.

In this article, we present advances in the original findings of a pragmatic utility concept analysis, from doctoral research, and those brought in a pre-print article, with a view to reflecting changes that improve conceptual knowledge and facilitate the theoretical construction and verification of specific SNC attributes^(15)^.

Findings from a pragmatic concept analysis can guide the future research decision, from qualitative approaches, for better description, clarification and design or indicate the possibility of advancing to quantitative studies, theory development and application^(16)^. Because of this, this article expands the interpretation of results currently available in the literature on the subject, investigating the process of conceptual maturation and being relevant for future research decisions on the subject^(15)^.

## OBJECTIVES

To analyze the SNC conceptual maturation from the perspective of pragmatic utility.

## METHODS

### Ethical aspects

Ethical issues related to research with human beings are not applicable, however, the principle of authorship rights was respected, with due source citation and indication^(17)^. The results and preliminary analyzes were deposited on the platform in pre-print format, by the same team of authors, without having received comments until the time of submission of manuscript to this journal. However, this manuscript is substantially different in content from most sections, incorporating blind reviewer recommendations and supporting our SNC concept’s interpretative evolution.

### Study design

This is a concept analysis study using the pragmatic utility method^(16,18-19)^, with writing guided by the Standards for Reporting Qualitative Research (SRQR) checklist.

Pragmatic utility is used to develop, clarify, delineate, compare, or correct concepts^(16)^. In it, epistemological, pragmatic, linguistic and logical principles are applied. The epistemological guide the assessment of the concept’s internal structure, which must be clear, distinct, internally consistent and differentiated from others. The pragmatist points out that the concept must be applicable to the world or operable. The linguistic indicates the appropriate use of the concept in context. The logician postulates conceptual coherence and the relationship with other concepts. These last three principles contribute to assessing the concept’s external structure^(16)^.

The present study analyzes the SNC concept’s internal structure through definitions, attributes, boundaries, preconditions and outcomes developed over time. In external assessment, existing limits with the competing concept are investigated: the NP^(16)^.

### Methodological procedures

We applied the method’s four operational stages: select the concept; elaborate analytical questions; comprehensively review the literature; and describe concept use in the scientific context^(16,18)^. In the first stage, the SNC concept was chosen, based on the diffuse scope of definitions and contextual changes over time already pointed out in the literature.

In the second stage, analysts constructed two analytical questions: what changes occurred in the SNC concept definition in nursing literature in Brazil over the years, indicating the conceptual design? What conceptual and contextual evidence indicated the concept maturation process?

### Data collection and organization

The third stage was to comprehensively review the literature. The method employed requires tracing the concept back as far as possible to what would presumably be its origins^(16)^. The aforementioned trail was obtained by combining a generic search using a search tool, access to literature data and selection of publications cited in the retrieved materials.

Initially, we performed a generic search with the Google Scholar^®^ tool, until October 2019, using the term “SNC”, without establishing a time limit. After adding the words, not descriptors, in English and Spanish, “*Sistematización de Enfermería*”, “Systematization of Nursing Care”, the authors checked 15,209 materials.

The inclusive extension of this search is compatible with the analytical principle of concept analysis methods, since it operates in a logic contrary to that of most literature review studies that tend towards synthesis and aggregation^(16)^. Concept analysis methods are not literature reviews per se, although they do review publications. They dispense with most of literature synthesis and summarization techniques required in empiricist-based reviews^(16)^, and do not apply the same quality criteria as the bibliographic sources of systematized reviews, especially when one considers that a concept is built evolutionarily by maturing and in the multiplicity of opinions, such as pragmatic utility^(16)^. Thus, in this research, Critical Theory’s philosophical foundation for material collection and selection for pragmatic utility analysis did not emphasize concept contextualism, as opposed to the observable evidence and universalization of the language of empiricist science^(19)^.

From the search to the preliminary analysis, 31 manuscripts that actually dealt with SNC were selected, excluding NP. Of these, 09 manuscripts were in the category regulations and professional consensus documents, namely: COFEN professional resolutions and synthesis documents of symposiums and congresses organized by ABEn. This procedure was useful to obtain old publications that could not be included in the literature databases and of other nature, essential to conceptual maturity analysis.

Later, in 2021, the researchers carried out a second search, which was structured in Latin American and Caribbean Literature in Health Sciences (LILACS), through the Virtual Health Library (VHL) portal.

Aligned descriptors were chosen due to the absence of the descriptor “Systematization of Nursing Care”, namely: (ti:(systematization OR organization OR *sistematizacao* OR *organizacao* OR *organizacion* OR *sistematizacion*)) AND (tw:(“nursing care” OR “*cuidado de enfermagem*” OR “*atencion de enfermeria*” OR “nursing assistance” OR “*assistencia de enfermagem*” OR “*assistencia de enfermeria*” OR “Nursing process” OR “*processos de enfermagem*” OR “*procesos de enfermeria*” OR “nursing assessment” OR “*avaliacao de enfermagem*” OR “*evaluacion de enfermeria*” OR nurse OR *enfermeiro* OR *enfermera*)) AND (tw:(“Health services” OR “*servicos de saude*” OR “*servicios de salud*” OR Health OR *saude* OR *salud* OR Hospitals OR *hospitais* OR *hospitales*)) AND (instance:”regional”) AND (db:(“LILACS”)).

The search limited to LILACS, although uncommon, is justified by the fact that publication on the subject occurs strictly in Brazilian journals. Terms translated from the SNC into other languages are used in translated versions of Brazilian articles. Also, the Google Scholar^®^ search had shown the absence of publication of relevant articles for concept analysis in foreign journals.

In reviewing the material, three researchers acted independently, using criteria from analytical questions to: investigate the concept’s temporal dynamics; identify how and when the concept was introduced; check its current usage; identify the components of the concept’s epistemological principle from definitions published over the years; check the understanding of cohesion or divergence between the authors; and estimate the plausibility criterion of the concept’s partial maturity hypothesis as a function of cohesion^(16)^.

The requirements judged as an, *a priori*, adequacy of material were: term comprehensive conceptualization; structured term definition; SNC concept purpose and/or use.

After preliminary analysis, three analysts selected 27 manuscripts from this second search. These were added to the 34 from the previous search, producing a corpus with 61 texts.

### Data analysis

Two authors involved in the documental and bibliographic textual analysis used the four principles of the method to outline the SNC concept, exploring understandings, ideas and uses of it since the emergence of conceptual bases in the literature^(16)^. This process was that of semantic decomposition and qualitative inference production. Two analytical questions were used: what changes occurred in the SNC concept definition in nursing literature in Brazil over the years, indicating the conceptual design? What conceptual and contextual evidence indicated the concept maturation process?

To describe the use of the concept in the scientific context, we considered: material temporal organization; analytical reading to extract conceptual and operational definitions; identification of conceptual attributes of uniqueness; historical correlation with the concept’s antecedent and consequent states; interpretation of contextual elements highlighted by the author of the material; judging concept maturation by verifying the absence of indistinction or overlapping, according to the pragmatic utility analysis strategy^(16)^.

## RESULTS

All manuscripts identified were by Brazilian authors and qualitative research predominated in the form of expert opinions, reflections or experience reports.

Analysts identified the temporal dynamics in four periods in the SNC concept maturing process, starting from the first analytical question and evidence of concept and context, with the second question. Chart 1 summarizes such findings by trait or timeline.

**Chart 1:** Concept description elements obtained from the answers to analytical questions, Rio de Janeiro, Rio de Janeiro, Brazil, 2022 (N=61)

Analytical questions	Concept description components
What changes occurred in the SNC concept definition in nursing literature in Brazil over the years, indicating the conceptual design?	1960 to 1990: organize, systematize and document nursing care using techniques and strategies (observation, interview, physical examination, clinical records and prescriptions) that were added to the NP^(20-25)^. 1990 to 2002: trend of merging the concepts of SNC and NP, being used as synonyms^(6,26-28)^. 2002 to 2009: publications seeking to clarify conceptual distinctions between SNC and NP^(6-7,29-30)^. As of 2009: COFEN Resolution 358/2009 makes a normative distinction between SNC and PE, indicating the relationship between both^(4,27-28)^.
What conceptual and contextual evidence indicated the concept maturation process?	Conceptual evidence: a) Conceptual consensualization: partial. SNC as a synonym for other concepts^(13,24,31-35)^. SNC as an independent concept^(6,30,36-39)^. b) Concept operationalization: NP operational elements, being presented as the SNC^(13,24-26,28,31-33)^. Contextual evidence: Term origin: academic teaching context in the 1960s/1970s, to provide systematic nursing practice, especially nurses^(20-21,24)^. Oriented towards “nursing care” in Brazilian hospital practice and presented in a publication by research professors^(26)^. Term development: initially, publicization of the NP^(20-21)^. Subsequently, conceptual confusion with the NP^(5,26-28,31-33)^. Finally, opening of a new demand for management and administration in nursing^(36,40)^. Boundaries: under construction, but still hampered by overlap with the mature NP concept. Limit of unique SNC attributes are linked to nursing management/administration^(36,38)^.

*Source: doi: https://doi.org/10.1590/SciELOPreprints.3344.*

The contextual and conceptual evidence were used as an interpretation basis for the SNC concept analysis in relation to epistemological, logical, linguistic and pragmatic principles, which are presented in Chart 2.

**Chart 2:** Systematization of Nursing Care concept components guided by the principles of pragmatic utility analysis, organized according to temporal change dynamics, Rio de Janeiro, Rio de Janeiro, Brazil, 2022 (N=61)

Principles	Time periods of conceptual change		
Epistemological	Decades from 1960 to 1990	From 2002 to 2009	From 2009 onwards
Attributes	Systematization of tasks, techniques and strategies for the NP^(24,27)^, work organization method^(40)^.	Nurse’s private activity^(13)^; NP stages^(13)^; organization into a system involving dynamically interrelated elements^(41)^.	Element related to organization of nursing care^(36)^; nursing management instrument^(34)^; work methodology understood as synonymous with NP^(34)^.
Preconditions	Unclear.	Scientific work method^(13)^; recognition of institutional reality; available resources; awareness of the entire nursing team; definition of the mission, philosophy and objectives of nursing service; nursing team intellectual (theoretical) preparation; theoretical framework definition; elaboration of NP instruments; practical preparation for the SNC implementation^(29)^.	Unclear in the literature of that time.
Outcomes	Organizes nurses’ actions through problem solving methodology^(23)^. From the perspective of being understood as synonymous with NP: better guidance for nursing work; offer of a humanized and individualized assistance; improvement in quality of care^(26)^.	Organizes professional work; makes possible the NP operationalization^(7)^; identifies health and disease situations^(13)^; improves quality of care^(1,33)^.	NP operationalization^(36)^; facilitation of the work process^(38)^.
Logical	SNC and NP: synonyms^(2 6-27)^	Two regulations with different perspectives to regulate the SNC implementation and implementation through the NP^(7,13)^	Different perspectives on the term.
Linguistic	Does not maintain limit when integrated into the NP concept^(24,26,42)^.	Refers to SNC and NP as distinct terms^(7-8)^; shares NP^(13)^ attributes; NP and SNC understood as synonyms^(28)^.	Differentiation movement of NP and SNC terms (SINADEN); NP and SNC, controversial and multifaceted terms, requiring clarification efforts; understood as a synonym of NP^(34)^; SNC and NP: different terms^(37-38)^.
Pragmatic	Operationalized through NP application^(2 3,29,4 3)^.	Operationalized through NP application^(7,13)^; operationalized by different ways of producing assistance that presuppose organization of conditions, material and human resources^(44)^.	Regulations that guide the operation of SNC through NP^(38,43,45-47)^.

*Source: doi: https://doi.org/10.1590/SciELOPreprints.3344.*

## DISCUSSION

We understand that the main contributions to the advancement of the subject in this conceptual analysis was to explore conditions of change in the idea of a SNC and how this was related to the NP. With originality, the research cuts temporal periods of SNC concept development, indicating the overlapping dynamics with the NP and subsequent construction of own management/organizational attributes.

The retreat of conceptual research to the 1960s was original and essential to trace the maturation process and delimit antecedent conditions for overlapping concepts that were not previously explored in the literature. Studies interested in SNC’s conceptual aspects use the interval between 1999 and 2002 for their analyses^(1-2,7)^, due to COFEN Resolutions^(7,13)^. The inclusion of the largest possible time window benefits from the conception that a concept design occurs when it can be separated from allied or competing concepts^(20)^.

From 1990 to 2002, the progressive change in the conceptual limits indicated a trend of merging the concepts of SNC and NP, which ultimately may have weakened the conceptual limits of the most internationally widespread concept, the NP. Thus, a terminological confusion was materialized in COFEN Resolution 272, with questionable application of the terms “process”, “systematization” and “Nursing Consultation”^(13)^. There was incorporation of NP stages as SNC components, being a basis for the future development of a concept for the latter.

We emphasize that we do not present SNC concept components by the principles of pragmatic utility analysis from 1990 to 2002, since, in this period, the fusion of SNC and NP concepts led to a convergence towards synonymy, making it reckless to interpret the principles of pragmatic analysis without the risk of expanding conceptual confusion.

The linguistic use of “systematization” and “systematize”, from 1960 to 1990, referred mainly to the actions, stages and procedures of organizing professional practice, especially by carrying out NP stages and procedures. Among the academics who proposed the term, the main interest was oriented towards organizing conditions for nurses’ clinical work in ways compatible with the NP. Thus, at that time, the systematization initiative would be for actions and behaviors organized by observation, physical examination, interview, diagnosis and care plan; these aggregates to the NP^(44)^. It can be said, roughly speaking, that there was no indication for the development of a specific SNC concept.

The literature indicates the academic community’s greater mobilization to seek conceptual distinctions between the terms SNC and NP, between 2002 and 2009, possibly due to terminological confusion exacerbated by Normative Resolutions^(6,13)^. The search for currents of thought indicates this contextual condition at the time^(6)^, considering the coexistence of terms associated with the SNC with different, similar or related semantics, such as Nursing Consultation, Nursing Care Methodology, Nursing Care Methodology, among others^(8)^. Furthermore, in those years, there were no conceptual analyses, at least published, to present attributes of the concept, contributing to the maintenance of different understandings. In view of this, we state that, from an epistemological perspective, the SNC concept’s internal structure and its external position in relation to other concepts were not well designed.

COFEN Resolution 358/2009 establishes in its considerations the evolution of Nursing Consultation and SNC concepts and that, based on these and other considerations, its content would provide for the SNC and NP implementation in public or private environments, in which professional nursing care takes place^(7)^. Questions were raised about whether the SNC and the NP would be pseudo-synonyms and about what traits or characteristics would be common to them or what semantics would distinguish them^(8)^.

We interpret that COFEN Resolution 358/2009, in the use of different terms and definitions for the SNC and NP, recognized the SNC as a concept or construct, being specifically aimed at organizing conditions for the systematic and deliberate execution of NP. This normative framework can be understood as an indicator of progress in concept maturity towards partial maturation.

The interpretation of contextual evidence supports the view of a historical maturation of the SNC concept. It results in the conjecture that the SNC started from a diffuse meaning of work systematics, which includes stages and clinical procedures inherent to the NP, moving towards a systemic organicist vision that is proper and necessary for the NP implantation and implementation. Thus, the systemic view seems to indicate a relationship between the SNC and the NP. One perspective would point to the NP as one of the ways or methods of systematizing care along with protocols and standardization of procedures^(28)^. The SNC would take place in two situations: in the organization of material and human conditions^(8)^ that would support the NP; and the necessary technical-legal competence^(28)^. Such organizational perspectives are corroborated by authors of a recent reflection study^(1)^.

It is likely that the General Systems Theory may have influenced the construction of a vision of integrated systems between a specific concept of systematization and NP. Also, the interconnection of concepts of SNC, NP and Resolution COFEN 358/2009 theories would indicate a systemic meaning^(7)^. Synthesis documents of discussions of ABEn events with themes related to the NP and SNC indicate efforts to expand and disseminate the understanding of concepts and relationships and integration between both^(3)^. In short, the systemic view would point to the SNC as an organized scheme of interdependent elements, based on principles related to each other, however different from the idea of process. It would also encompass specific methods, actions, norms and procedures based on a theory for carrying out the process^(48)^.

Considering the linguistic principle, using SNC and NP as pseudo-synonyms, although reduced in publications, still exists and reinforces confusion in the language spoken in professional daily life and vice versa. The consequence of these inaccuracies in disciplinary and academic language would be to make it difficult for the concept to mature in the indication of more precise operational elements^(16)^.

We understand that a new SNC concept was generated with management characteristics and there is a growing professional effort to improve the understanding of its relations with the NP. Applying the logical principle requires the identification of relationships between concepts and their attributes^(16)^. However, the emergence of a new concept does not follow an uncritical construction. Pragmatic utility analysis’ philosophical basis is the paradigm of critical theory, which indicates the view of the concept as probabilistic, i.e., SNC and NP attributes will always be perceived by their similarities, by the observance of strict criteria of their own entities and by the value established in their usefulness for the subject^(18)^. By the principles of pragmatic utility, researchers’ efforts to investigate SNC’s singular aspects are understood as an essential requirement for its conceptual maturation^(1-2,11)^. The investigation in the country of systematization and NP themes previously directed to the understanding of terms and the definition of implementation strategies^(49-50)^ have received new advances to create conceptual models integrating the SNC and the NP, which can specify the relationships in a descriptive and explanatory way^(1-2)^.

By the pragmatic principle, we consider that there was a tacit construction of the SNC concept to deal with the practice organization phenomenon, which was analyzed in this manuscript. It started with a vague idea of systematizing the singular practice of nurses in relation to other practitioners of care through using the NP; however, progressively, the implementation of a NP created the need to restructure organizational aspects and staff training, which would lead to the recognition of a specific SNC entity. As a concept or construct, the SNC seems to be better defined nowadays for management and organizational attributes, either recognizing it as a concept originated in nursing management^(51)^ or indicating its dependence on management^(2)^.

Faced with the interpretation of SNC conceptual maturation’s complexity, we propose a definition of systemic anchorage: ‘SNC is an organizational work method based on theoretical-scientific elements, capable of providing conditions for NP operationalization as well as being justified and improved by its results’.

The interdependence of SNC and NP stems from this definition. The SNC, as a method, operates as an antecedent or condition of pre-established organizational type, to model a set of actions that facilitate NP operationalization. On the other hand, from the historical point of view, the SNC is a sequential, terminological and conceptual consequent of the “NP” concept and term. Somehow, both concepts retain a causality of a recursive nature that, although relevant for a true systematization, can still generate difficulties in conceptual understanding.

It seems essential to us to recognize the SNC as a construct specific to the organizational type and different from the NP, given the existence of multidisciplinary care systems that do not primarily use the NP, such as the Singular Therapeutic Project (PTS - *Projeto Terapêutico Singular*). Thus, the SNC, in its organizational perspective, will tend to support its own decisions to achieve a PTS and suffer particular interference arising from this multidisciplinary strategy.

Relating the SNC to the NP is the central aspect constructed for decades by the area, especially in the perspective that the NP confers scientific rationality to highlight the specificity of subject/profession’s know-how^(10)^. However, the organizational meaning of nursing practice involves situations that do not identify and are exclusive to the subject in health care, which makes it impossible to circumscribe all professional action to the NP. This issue demands an advance of reflections for the field of nursing and health management and invites to this debate researchers who are not exclusively those interested in NP and standardized language systems.

Thus, the philosophical foundations and methodological procedures of pragmatic utility analysis seem to have been adequate to verify conceptual maturation concepts over the more than 40 years since the introduction of original ideas of a “systematization” for nursing care. However, the fragile consensus on definitions and structural elements of SNC reflects a process that is still in partial maturity^(20)^.

### Study limitations

The authors recognize a study limitation in offering objectifiable elements that are essential to build an operational definition for the SNC.

It is possible to understand the substantial challenges of currently producing research that validates SNC strategies in the sense of an organizational or management system, before resolving conceptual confusions. Also, concept elements are under construction in the implantation and implementation in different health units in the country, influenced by the absence of referential, attributes and robust constitutive definitions about the concept. Based on this, the authors recommend two guidelines for future studies: the first in relation to developing conceptual and empirical models for the SNC that would serve as a reference structure; the second in relation to exploratory and analytical studies that provide evidence of the application of individualized initiatives and of targeted interactions with the NP.

### Contributions to nursing

This analysis contributes to the field of nursing, by providing evidence on different periods of SNC concept maturation, verifying its current point of partial maturity and proposing a general definition. In this regard, this study enables a better understanding of the phenomenon that the SNC concept represents in Brazilian nursing practice.

The study presents a conceptual outline and clarification without abandoning the historical or temporal context in which the concept was developed. The findings of this research, in addition to contributing to current scientific debate and entities representing the professional category of nursing, give room for a possible and necessary adequacy of language and operationalization in the training and assistance spaces. The study can also serve as a reference for changes in policies and regulations that deal with and regulate the SNC.

## CONCLUSIONS

The SNC concept is still a partially mature concept, presenting multiple definitions, being operationalized in uncertain links with other concepts, implying the current state of knowledge in an elusive and confusing concept.

Analysis by the pragmatic utility method was useful to verify the SNC concept maturation process in four time periods. It also helped in verifying the process’ conceptual maturation and characteristics and in the outlining of a definition that could be useful for its operationalization in the context.

## Data Availability

https://doi.org/10.1590/SciELOPreprints.3344

## References

[1] Santos GLA, Sousa AR, Félix NDC, Cavalcante LB, Valadares GV. (2021). Implicações da Sistematização da Assistência de Enfermagem na prática profissional brasileira. Rev Esc Enferm USP.

[2] Santos GL, Valadares GVV. (2022). Sistematização da Assistência de Enfermagem: buscando contornos teóricos, definitórios e diferenciadores. Rev Esc Enferm USP.

[3] Garcia TR, Nóbrega MML. (2019). Simpósio Nacional de Diagnóstico de Enfermagem: building a knowledge field for Nursing. Rev Bras Enferm.

[4] Conselho Federal de Enfermagem (Cofen) (2021). Portaria n°1226 de 8 de outubro de 2021.

[5] Virgínio NA, Nóbrega MML. (2004). Sistematização da Assistência de Enfermagem: revisão da literatura. Rev Ciênc Saúde Nova Esperança [Internet].

[6] Fuly PSC, Leite JL, Lima SBS. (2008). Concepts associated to systematization of nursing care in Brazilian journals. Rev Bras Enferm.

[7] Conselho Federal de Enfermagem (Cofen) (2009). Dispõe sobre a Sistematização da Assistência de Enfermagem e a implementação do Processo de Enfermagem em ambientes, públicos ou privados, em que ocorre o cuidado profissional de Enfermagem, e dá outras providências.

[8] Garcia TR, Nóbrega MML. (2009). Systematization of nursing care: is there agreement on the concept. Rev Eletrônica Enferm.

[9] Garcia TR. (2016). Systematization of nursing care: substantive aspect of the professional practice. Esc Anna Nery.

[10] Gutiérrez MGR, Morais SCR. (2017). Systematization of nursing care and the formation of professional identity. Rev Bras Enferm.

[11] Barros ALBL, Lucena AF, Morais SCRV, Brandão MAG, Almeida MA, Cubas MR (2022). Processo de Enfermagem no contexto brasileiro: reflexão sobre seu conceito e legislação. Rev Bras Enferm.

[12] Boaventura AP, Santos PA, Duran ECM. (2017). Conocimiento teórico-práctico del Enfermero del Proceso de Enfermería y Sistematización de Enfermería. Enfermería Glob.

[13] Conselho Federal de Enfermagem (Cofen) (2002). Dispõe sobre a Sistematização da Assistência de Enfermagem - SAE - nas Instituições de Saúde Brasileiras.

[14] Slatyer S, Coventry L, Twigg D, Davis S. (2016). Professional practice models for nursing: a review of the literature and synthesis of key components. J Nurs Manag.

[15] Souza JF. (2021). Análise do conceito de Sistematização da Assistência de Enfermagem: perspectiva da utilidade pragmática.

[16] Morse JM. (2016). New.

[17] Presidência da República (BR) (2018). Lei nº 13.709, de 14 de agosto de 2018. Lei Geral de Proteção de Dados Pessoais (LGPD).

[18] Weaver K, Mitcham C. (2008). Nursing concept analysis in North America: state of the art. Nurs Philos.

[19] Hawkins SF, Morse J. (2014). The Praxis of Courage as a Foundation for Care. J Nurs Scholarsh.

[20] Horta WA. (1967). Considerações sobre o diagnóstico de enfermagem. Rev Bras Enferm.

[21] Horta WA. (1971). A metodologia do processo de enfermagem. Rev Bras Enferm.

[22] Carvalho V. (1972). A problemática do diagnóstico de enfermagem. Rev Bras Enferm.

[23] Iwanow Cianciarullo T, Sumie Koizumi M, Áurea Quintela Fernandes R. (1974). Prescrição de enfermagem: experiências de sua aplicação em hospital particular. Rev Bras Enferm.

[24] Paula NS, Gonçalves MMC, Cianciarullo TI, Fernandes RÁQ, Friedlander MR, Campedelli MC (1978). Processo de Enfermagem orientado para os problemas do paciente: iniciação de ensino de fundamentos em enfermagem. Rev Bras Enferm.

[25] Cruz DALM, Ribeiro FG, Dutra VO, Caracciolo LT. (1987). Sistematização da Assistência de Enfermagem em uma área de recuperação da saúde. Rev Esc Enferm USP.

[26] María VLR, Dias AMC, Shiotsu CH, Farias FAC. (1987). Sistematização da assistência de enfermagem no Instituto “Dante Pazzanese” de Cardiologia: relato de experiência. Rev Esc Enferm USP.

[27] Tsunechiro MA, Carvalho DV, Posso MBS, Elsas BX, Lui MC, Stefanelli MC. (1983). Instrumento para análise de periódico de enfermagem. Rev Esc Enferm USP.

[28] Venturini DA, Matsuda LM, Waidman MAP. (2009). Produção científica brasileira sobre sistematização da assistência de enfermagem. Ciên Cuid Saúde.

[29] Hermida PMV, Araújo IEM. (2006). Sistematização da assistência de enfermagem: subsídsios para implantação. Rev Bras Enferm.

[30] Carvalho EC, Bachion MM, Dalri MCB, Jesus CAC. (2007). Obstáculos para a implementação do processo de enfermagem no Brasil. Rev Enferm UFPE.

[31] Varela GDC, Fernandes SCDA. (2013). Conhecimentos e práticas sobre a sistematização da assistência de enfermagem na estratégia saúdem da família. Cogitare Enferm.

[32] Andrade JS, Vieira MJ. (2005). Prática assistencial de enfermagem: problemas, perspectivas e necessidade de sistematização. Revista brasileira de enfermagem. Rev Bras Enferm.

[33] Nascimento KC, Backes DS, Koerich MS, Erdmann AL. (2008). Sistematização da assistência de enfermagem: vislumbrando um cuidado interativo, complementar e multiprofissional. Rev Esc Enferm USP.

[34] Cruz ADMP, Almeida MA. (2010). Competências na formação de Técnicos de Enfermagem para implementar a Sistematização da Assistência de Enfermagem. Rev Esc Enferm USP.

[35] Reppetto MA, Souza MF. (2005). Avaliação da realização e do registro da Sistematização da Assistência de enfermagem (SAE) em um hospital universitário. Rev Bras Enferm.

[36] Soares MI, Resck ZMR, Camelo SHH, Terra FS. (2016). Gerenciamento de recursos humanos e sua interface na sistematização da assistência de enfermagem. Enferm Glob.

[37] Castro RR, Alvino ALFN, Rouberte ESC, Moreira RP, Oliveira RL. (2016). Compreensões e desafios acerca da sistematização da assistência de enfermagem. Rev Enferm UERJ.

[38] Torres E, Christovam BP, Fuly PCS, Silvino ZR, Andrade M. (2011). Sistematização da assistência de enfermagem como ferramenta da gerência do cuidado: estudo de caso. Esc Anna Nery.

[39] Dell'Acqua MCQ, Miyadahira AMK. (2000). Processo de enfermagem: fatores que dificultam e os que facilitam o ensino. Rev Esc Enferm USP.

[40] Kurcgant P. (1991). A prática da administração em enfermagem. Rev Esc Enferm USP.

[41] Crossetti MGO, Barros ALBL, Brandão MAG, Nóbrega MML, Corbellini VL. (2007). Painel: Sistematização da Assistência de Enfermagem. Enfermagem Atual.

[42] Luckesi MAV, Amorim MJAB, Silva NF, Nuñes RS. (1978). Aplicação do processo de enfermagem no hospital Ana Nery: relato de uma experiência. Rev Bras Enferm.

[43] Conselho Federal de Enfermagem (Cofen) (2016). Atualiza as normas técnicas para Anotação de responsabilidade Técnica pelo serviço de enfermagem e define as atribuições do Enfermeiro Responsável Técnico.

[44] Horta WA. (1974). Enfermagem: teorias, conceitos, princípios e processo. Rev Esc Enferm USP.

[45] Conselho Federal de Enfermagem (Cofen) (2012). Dispõe sobre o registro das ações profissionais no prontuário do paciente, e em outros documentos próprios da enfermagem, independente do meio de suportetradicional ou eletrônico.

[46] Conselho Federal de Enfermagem (Cofen) (2017). Estabelece os parâmetros mínimos para dimensionar o quantitativo de profissionais das diferentes categorias de enfermagem para os serviços/locais em que são realizadas atividades de enfermagem.

[47] Conselho Federal de Enfermagem (BR) (2017). Aprova o novo código de ética dos profissionais de enfermagem.

[48] Daniel LF. (1981). A Enfermagem Planejada.

[49] Kletemberg DF, Siqueira MD, Mantovani MF. (2006). Uma história do processo de enfermagem nas publicações da Revista Brasileira de Enfermagem no período 1960-1986. Esc Anna Nery.

[50] Paula NS, Farias GM, Araújo TL, Takahashi OC. (1984). Assistência de enfermagem sistematizada: experiência de aprendizado. Rev Bras Enferm.

[51] Santana RF. (2019). Sistematização da assistência de enfermagem, uma invenção brasileira?. Rev Enferm Atenc Saúde.

